# Alginate Oligosaccharide Alleviated Cisplatin-Induced Kidney Oxidative Stress *via Lactobacillus* Genus-FAHFAs-Nrf2 Axis in Mice

**DOI:** 10.3389/fimmu.2022.857242

**Published:** 2022-04-01

**Authors:** Yubing Zhang, Song Qin, Yipeng Song, Jingyi Yuan, Shanliang Hu, Min Chen, Lili Li

**Affiliations:** ^1^ Yantai Institute of Coastal Zone Research, Chinese Academy of Sciences, Yantai, China; ^2^ College of Life Sciences, Yantai University, Yantai, China; ^3^ Center for Ocean Mega-Science, Chinese Academy of Sciences, Qingdao, China; ^4^ Department of Radiotherapy, Yantai Yuhuangding Hospital, Yantai, China

**Keywords:** alginate oligosaccharide, cisplatin, gut microbiota, metabolomics, FAHFAs, Nrf2, oxidative stress, inflammation

## Abstract

Alginate oligosaccharide is the depolymerized product of alginate, a natural extract of brown algae, which is associated with beneficial health effects. Here, we aimed to investigate the mechanism *via* which alginate oligosaccharides improve kidney oxidative damage and liver inflammation induced by cisplatin chemotherapy *via* the gut microbiota. C57BL/6J mice were treated with cisplatin were administered alginate oligosaccharide *via* gavage for 3 weeks. Compared to that observed in the cisplatin chemotherapy group without intragastric administration of alginate oligosaccharide, liver inflammation improved in the alginate oligosaccharide group, indicated by reduction in lipopolysaccharide and interleukin-1*β* (IL-1*β*) levels. This was accompanied by improvement in the oxidative stress of mice kidneys, indicated by the increase in the levels of superoxide dismutase (SOD), catalase (CAT) and nuclear NF-E2-related factor 2 (Nrf2) in renal tissue, and reduction in the levels of malondialdehyde (MDA) in renal tissue and serum creatinine (Cr) to the levels of the normal control group. Alginate oligosaccharide intervention increased the concentration of fatty acid esters of hydroxy fatty acids (FAHFAs). Alginate oligosaccharide regulated the composition of the intestinal microbial community and promoted *Lactobacillus* stains, such as *Lactobacillus johnsonii* and *Lactobacillus reuteri*. Spearman analysis showed that 5 members of FAHFAs concentrations were positively correlated with *Lactobacillus johnsonii* and *Lactobacillus reuteri* abundance. We observed that alginate oligosaccharide increased FAHFAs producing-related bacterial abundance and FAHFAs levels, enhanced the levels of SOD and CAT in kidney tissue, and reduced the levels of MDA *via* activating Nrf2, thereby ameliorating the renal redox injury caused by cisplatin chemotherapy.

## Introduction

Cisplatin is a highly effective antineoplastic drug used for treating many solid tumors. However, its use is limited by its adverse effects on normal tissues. Acute kidney injury (AKI) is the most common side effect of cisplatin (1), a widely used chemotherapeutic drug. Currently, specific drugs that can continuously reduce or prevent cisplatin-induced AKI are not available (2). Oxidative stress does not only play a pivotal role in the progression of inflammatory diseases, but is also involved in the development of cancer. Therefore, development of new strategy to improve the local inflammation and oxidative stress induced by cisplatin is urgently required (3). The application of natural substances to reduce the side effects of cisplatin chemotherapy is an important and evolving subject in cancer treatment.

According to the China Fisheries Statistics Yearbook (2018), the aquaculture output of kelp in 2017 has reached 66.73% of the total aquaculture output of algae, and kelp is the most important aquaculture algae. Alginate oligosaccharide is the degradation product of alginate, the main extracted product of kelp and has attractive pharmaceutical properties (4). Studies have shown that alginate oligosaccharide improved cellular oxidative stress in neuron-like PC12 cells (5). In a weaned pig model, alginate oligosaccharide acted against intestinal barrier injury and inflammation (6). Alginate oligosaccharides have recently been shown to rescue busulfan-induced mucositis (4), save sperm motility (7) and rescue of male fertility (8) by altering the gut microbiota. Our previous studies also confirmed that the role of alginate oligosaccharide in the regulation of lipid metabolism and the improvement of inflammation was related to the regulation of the gut microbiota composition (9). These reports confirmed the potential of alginate oligosaccharide to improve the side effects of cisplatin therapy, especially renal oxidative stress and liver inflammation.

The molecular mechanism of gut microbiota controlling immunity has just been explored in recent years (10). However, reports on the improvement of cisplatin-induced inflammation and redox injury by alginate oligosaccharide *via* the gut microbiota are lacking. Therefore, we studied the therapeutic mechanism *via* which alginate oligosaccharide prevents cisplatin-induced inflammation and oxidative stress. Our observations indicated that alginate oligosaccharide may be combined with intestinal bacteria and their metabolites for treating AKI in future.

## Materials and Methods

### Animals and Diets

Alginate oligosaccharide [degree of polymerization=1–4, see previous article for structure (9)]. Cisplatin (*cis*-diammineplatinum dichloride CAS number: 15663-27-1) was purchased from Shanghai Macklin Biochemical Co., Ltd. (Shanghai, China). SPF C57BL/6J mice (six-week-old, male) were purchased from Jinan Pengyue Experimental Animal Technology Co., Ltd. (Shandong, China). Mice were fed under environmentally controlled conditions (humidity, 40−60%; temperature, 20−25°C; 12 h/12 h light/dark cycles). All mice were acclimatized for one week prior to formal experiments. Mice were randomly divided into three groups with 10 mice per group. The group without cisplatin chemotherapy, which was treated with phosphate buffered saline (PBS), was named NC; the group with PBS intervention after cisplatin chemotherapy (two weeks later, 10mg/kg cisplatin + PBS buffer solution 0.2 ml was injected intraperitoneally (11, 12); the other groups were only injected with PBS 0.2 ml) treatment was named CIS; the group with alginate oligosaccharide intervention after chemotherapy was named AO. Alginate oligosaccharide (5 mg/ml) (13) in PBS or PBS alone was gavage administered to mice in the AO group (0.2 ml), CIS group (0.2 ml), and NC group (0.2 ml) for three weeks. The study was conducted according to the guidelines of the Declaration of Helsinki. Approval Form for the Ethics committee of the Yantai Yuhuangding Hospital (Approval NO. 403-2019). The specific experimental design process is shown in **Table 1**. The feces were collected in a sterilized single cage per mouse. The fresh feces were immediately frozen in liquid nitrogen. At the end of the experiment, after anesthesia with pentobarbital sodium 50 mg/kg, then the mice were killed by decapitation method. After the mice died, the liver and kidney were immediately collected, the blood was washed off with PBS, and immediately frozen in liquid nitrogen.

**Table 1:** The experimental design protocol.

Day	NC group	CIS group	AO group
-7	Mice arrived and acclimatized for one week		
0-21	Daily gavage with PBS		Daily gavage with PBS + AO (50 mg/kg, 0.2 ml)
14	PBS intraperitoneal injection	PBS + CIS intraperitoneal injection (10 mg/kg, 0.2 ml)	
19-20	Feces were collected for gut microbiota and microbiome analyses		
21	The mice were killed by cervical dislocation and the liver and kidney tissues were obtained		

NC Group, Normal control group; CIS Group, Cisplatin treated group; AO Group, Alginate oligosaccharide treated group; PBS, Phosphate buffer saline.

### Enzyme Linked Immunosorbent Assay (ELISA) Analysis

The levels of serum creatinine (Cr) catalog number ML037726, kidney tissue superoxide dismutase (SOD) catalog number ML643059, catalase (CAT) catalog number ML037752, malondialdehyde (MDA) catalog number ML016824, and NF-E2-related factor 2 (Nrf2) catalog number ML037744 were determined according to the manufacturer’s instructions using ELISA kits from Mlbio Company (Shanghai, China). The levels of liver tissue interleukin-1*β* (IL-1*β*) using ELISA kits from R&D Systems catalog number MLB00C (Minneapolis, USA), the levels of liver tissue lipopolysaccharide (LPS) catalog number ML037221 were using ELISA kits from Mlbio Company (Shanghai, China). The nuclear protein of kidney tissue was extracted using the nuclear and cytoplasmic protein extraction kit from Labgic Technology Co., Ltd. (Beijing, China).

### Microbiota Analysis

Microbiota samples were extracted from feces using QIAamp DNA stool kit (QIAGEN Inc., Germantown, MD, USA). The variable regions V3-V4 of the 16S rRNA genes were amplified using the primers 515F (′GTGCCAGCMGCCGCGGTAA′) and 806R (′GGACTACHVGGGTWTCTAAT′). The purified amplicons were analyzed using paired-end sequencing on the Illumina NovaSeq system (San Diego, CA, USA). The sequences were clustered into operational taxonomic units (OTUs) with 97% identity. The Unweighted UniFrac distance matrix was used to perform ANOSIM statistical tests in QIIME (Version 1.9.1). The original raw sequence data of the mouse gut microbiota had been deposited in NCBI Sequence Read Archive (SRA) under the accession number PRJNA779197.

### Metabolome Analysis

The metabolome was analyzed using Exion LC (SCIEX) equipped with mass spectrometer of QTRAP6500+ (SCIEX, Framingham, USA). Samples were detected using BEH C8 column (1.7 *μ*m × 2.1 mm × 100 mm, Waters) with flow rate of 0.35 ml/min in positive electrospray ionization mode. The mobile phases used were 0.1% (*v*/*v*) formic acid-water and 0.1% (*v*/*v*) formic acid-acetonitrile. Samples were detected using the HSS T3 column (3.5 *μ*m × 4.6 mm × 250 mm, Waters) with flow rate of 0.35 ml/min in negative electrospray ionization mode. The mobile phase was 6.5 mmol/L ammonium bicarbonate and 6.5 mmol/L ammonium bicarbonate-95% (*v*/*v*) methanol. Metabolites were further analyzed according to the previous method.

### Statistical Analysis

Data were analyzed using the IBM SPSS Statistics version 26.0 (International Business Machines Corporation, USA). T-test was used to evaluate the difference between two groups. *p<*0.05 was considered significantly different.

## Results

### Analysis of Differential Biochemical Indexes After Alginate Oligosaccharide Treatment

**Figure 1:**
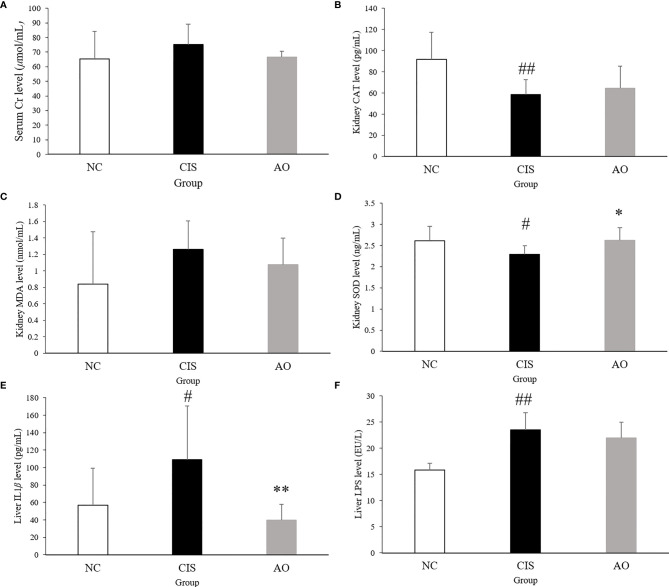
Biochemical parameters and oxidative stress analysis in renal and hepatic tissues. **(A)** serum Cr level; **(B)** kidney CAT level; **(C)** kidney MDA level; **(D)** kidney SOD level; **(E)** liver IL1β level; **(F)** live LPS level. n=10 for the AO and NC groups, n=7 for the CIS group (three mice in the CIS group died before euthanasia.) The significance marker on AO indicates AO vs. CIS (*p < 0.05, **p < 0.01); the significance marker on CIS indicates CIS vs. NC (^#^ *p* < 0.05, ^##^ *p* < 0.01).

Our results showed that serum Cr, liver IL-1*β* and LPS levels, and kidney MDA level decreased after alginate oligosaccharide treatment. Kidney CAT and SOD level increased, indicating that alginate oligosaccharide treatment reduced cisplatin chemotherapy-induced damage to renal antioxidant activity (**Figure 1**). The intervention of alginate oligosaccharide elicited therapeutic effects on cisplatin chemotherapy-induced liver inflammation. Alginate oligosaccharide exerted a certain reparative effect on cisplatin-induced damage to kidney redox capacity.

### Effects of Cisplatin and Cisplatin With Alginate Oligosaccharide on Microbial Communities

The changes in bacterial communities after prebiotic analysis were analyzed using nonmetric multidimensional scaling, which demonstrated a distinct clustering of microbiota composition for each group. ANOSIM analysis further proved that the gut microbial communities were significantly modulated in the AO group (*p*=0.001).

**Figure 2:**
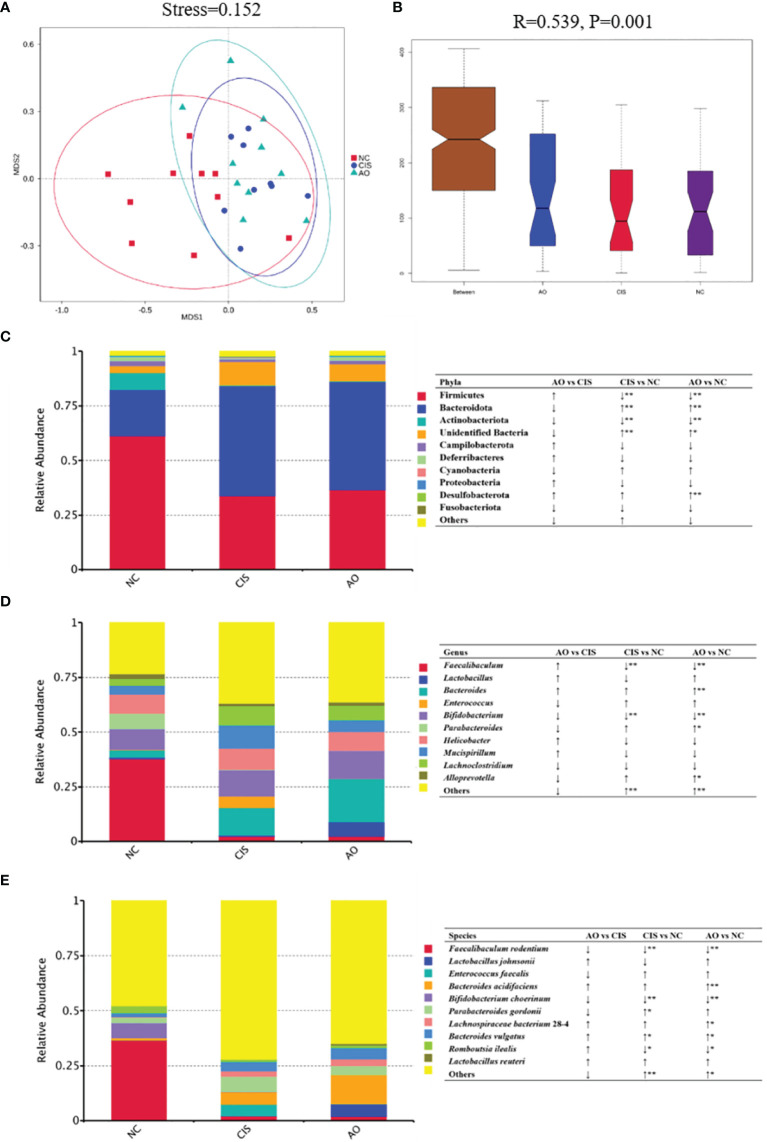
Gut microbiota analysis. **(A)** Non-metric dimensional scaling (NMDS) plots; **(B)** ANOSIM analysis of the significance of separation between the NC, CIS, and AO groups; **(C)** relative abundance of the top 10 phyla; **(D)** relative abundance of top ten genus; **(E)** relative abundance of top ten species; n=10 for the AO and NC groups, n=9 for the CIS group (one mouse in the CIS group died while collecting feces). NC, normal control; CIS, cisplatin; AO, cisplatin with alginate oligosaccharides (*p < 0.05, **p < 0.01; ↑, upregulation; ↓, downregulation).

Cisplatin and cisplatin with alginate oligosaccharide intervention caused distinct shifts in the bacterial genera (**Figure 2**). At the phylum level, the abundance of Firmicutes and Actinobacteriota decreased significantly (*p<*0.01) and the abundance of Bacteroidota increased significantly (*p<*0.01) in the AO and CIS groups compared to that in the NC group. At the genus level, the abundance of *Faecalibaculum* and *Bifidobacterium* decreased significantly (*p<*0.01), while that of *Bacteroidetes* increased significantly (*p<*0.01) in the AO and CIS groups compared to that in the NC group. Although not statistically significant (*p*>0.05), the treatment with alginate oligosaccharide not only reversed the cisplatin-induced decrease in abundance of *Lactobacillus*, but also increased it above the level of the NC group. At the species levels, the abundance of *Faecalibaculum rodentium* and *Bifidobacterium choerinum* decreased significantly (*p<*0.01), that of *Romboutsia ilealis* decreased significantly (*p<*0.05), and that of *Bacteroides vulgatus* increased significantly (*p<*0.05) in the AO and CIS groups compared to that in the NC group. The species level data indicated that the elevated abundance of *Lactobacillus* was mainly due to increase in the abundance of *Lactobacillus johnsonii*.

### Effects of Cisplatin and Cisplatin With Alginate Oligosaccharide on Metabolites in the Feces

**Figure 3:**
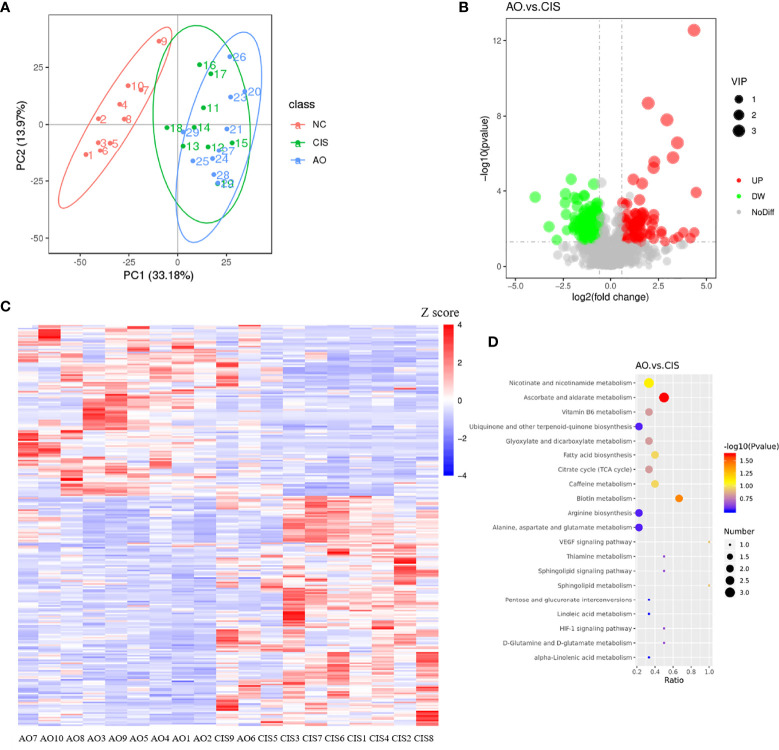
Metabolomics analysis in feces. **(A)** principal components analysis (PCA) of all metabolic pathways in three groups; **(B)** volcano map of differential metabolites between AO group and CIS group; **(C)** heat map of differential metabolites between AO group and CIS group; **(D)** KEGG analysis of differential metabolites between AO and CIS groups. n=10 for the AO and CIS groups, n=9 for the CIS group (one mouse in the CIS group died while collecting feces). Z score value represents the normalized abundance. VIP value represents correlation coefficient.

After the intervention, we analyzed the metabolites in the feces of mice. The different metabolite volcano plots between AO group and CIS group are shown in **Figures 3A, B**. In total, 267 significantly different metabolites were identified in AO vs. CIS (*p<*0.05), among which 125 metabolites showed highly significant differences (*p<*0.01); the abundance of 53 of these 125 metabolites increased significantly, whereas that of 72 metabolites decreased significantly. All significantly different metabolite heat maps in AO vs. CIS are shown in **Figure 3C**.

### Cisplatin and Cisplatin With Alginate Oligosaccharide Intervention Caused Distinct Shifts in the Different Metabolite-Related Metabolic Pathways

The analysis of the different metabolite-related metabolic pathways between each group is shown in **Figure 3D**. According to our Kyoto Encyclopedia of Genes and Genomes (KEGG) pathway analysis, ascorbate and aldarate metabolism and biotin metabolism differed significantly in AO vs. CIS (*p<*0.05).

### Analysis of Differential Metabolites and the Microbiome After Alginate Oligosaccharide Treatment

**Figure 4:**
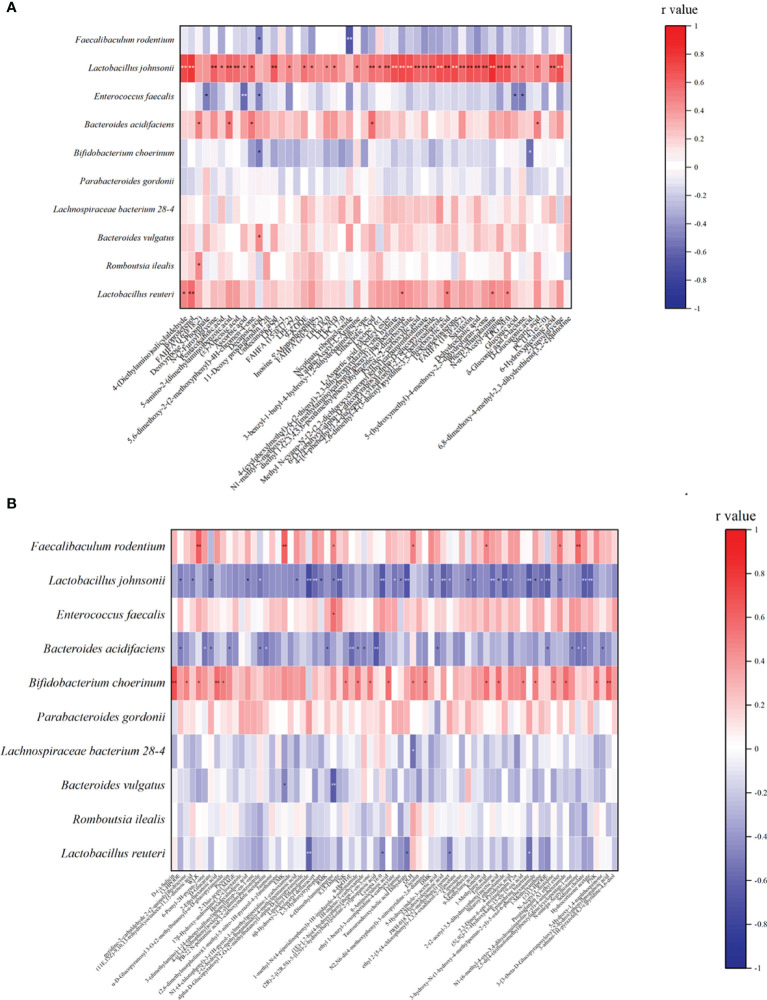
Spearman correlation analysis between the most significant difference (*p<*0.01) metabolites and the top 10 microbial species with the highest abundance. **(A)** The most significant increased metabolites (*p<*0.01) and the top 10 microbial species with the highest abundance between AO and CIS groups; **(B)** the most significant decreased metabolites (*p<*0.01) with the top 10 microbial species with the highest abundance between AO and CIS groups. *p < 0.05, **p < 0.01.

We analyzed the relationship between alginate oligosaccharide intervention with different metabolites and species. We analyzed the Spearman correlation between all significantly up-regulated metabolites in the AO and CIS groups and the top 10 species, and the results are shown in the **Figure 4A**. We found that *Faecalibaculum rodentium* correlated significantly negatively with decanoic acid (*p<*0.05) and nicotinate ribonucleoside (*p<*0.01), *Lactobacillus johnsonii* correlated significantly positively with 39 highly significantly up-regulated metabolites, *Enterococcus faecalis* correlated significantly negatively with 5 highly significantly up-regulated metabolites, *Bacteroides acidifaciens* correlated significantly positively with 5 highly significantly up-regulated metabolites, *Bifidobacterium choerinum* correlated significantly negatively with decanoic acid (*p<*0.05) and D-glucuronic acid (*p<*0.05), *Bacteroides vulgatus* correlated significantly negatively with decanoic acid (*p<*0.05), *Romboutsia ilealis* correlated significantly positively with FAHFA (16:0/18:2) (*p<*0.05), and *Lactobacillus reuteri* correlated significantly positively with 6 highly significantly up-regulated metabolites.

We analyzed the Spearman correlation between all metabolites significantly down-regulated in the AO and CIS groups and the top 10 strains, the results of which are shown in the **Figure 4B**. We found that *Faecalibaculum rodentium* correlated significantly positively with 7 highly significantly down-regulated metabolites, *Lactobacillus johnsonii* correlated significantly negatively with 31 highly significantly down-regulated metabolites, *Enterococcus faecalis* correlated significantly positively with 6-(dimethylamino) purine, *Bacteroides acidifaciens* correlated significantly negatively with 17 highly significantly down-regulated metabolites, *Bifidobacterium choerinum* correlated significantly positively with 19 highly significantly down-regulated metabolites, *Lachnospiraceae bacterium* 28-4 correlated significantly positively with QLH, *Bacteroides vulgatus* correlated significantly positively with EMK and 6-(Dimethylamino) purine, and *Lactobacillus reuteri* correlated significantly negatively with 5 highly significantly down-regulated metabolites.

### AO Significantly Increased the Levels of Intestinal FAHFAs Relative to the Cisplatin

**Figure 5:**
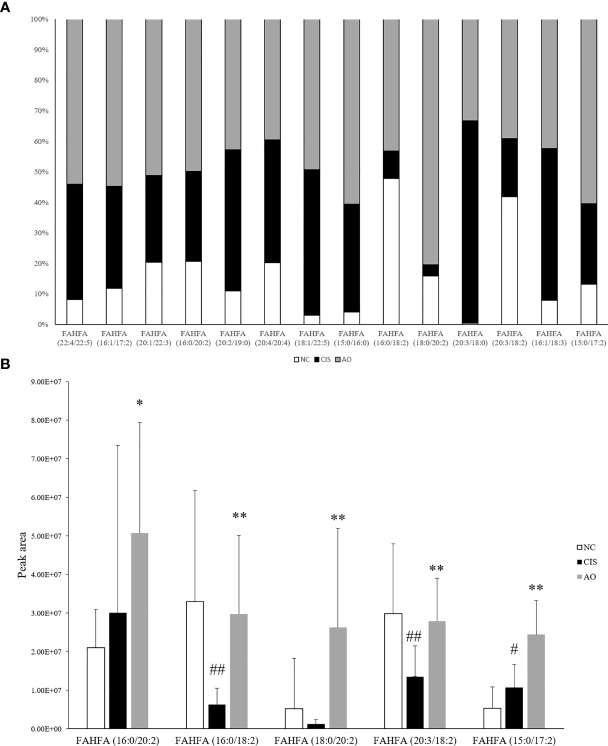
FAHFAs levels in feces. **(A)** Comparison diagram of relative content of all we matched FAHFAs; only FAHFA (20:2/19:0), FAHFA (20:4/20:4), FAHFA (20:3/18:0) and FAHFA (16:1/18:3) were decreased in AO vs. CIS. but those FAHFAs were no significant difference between AO and CIS groups (p>0.05). **(B)** Relative content bar graph showing significant differences in AO vs. CIS FAHFAs; the significance marker on AO indicates AO vs. CIS (*p<0.05, **p<0.01), the significance marker on CIS indicates CIS vs. NC (^#^ *p<*0.05, ^##^ *p<*0.01).

**Figure 6:**
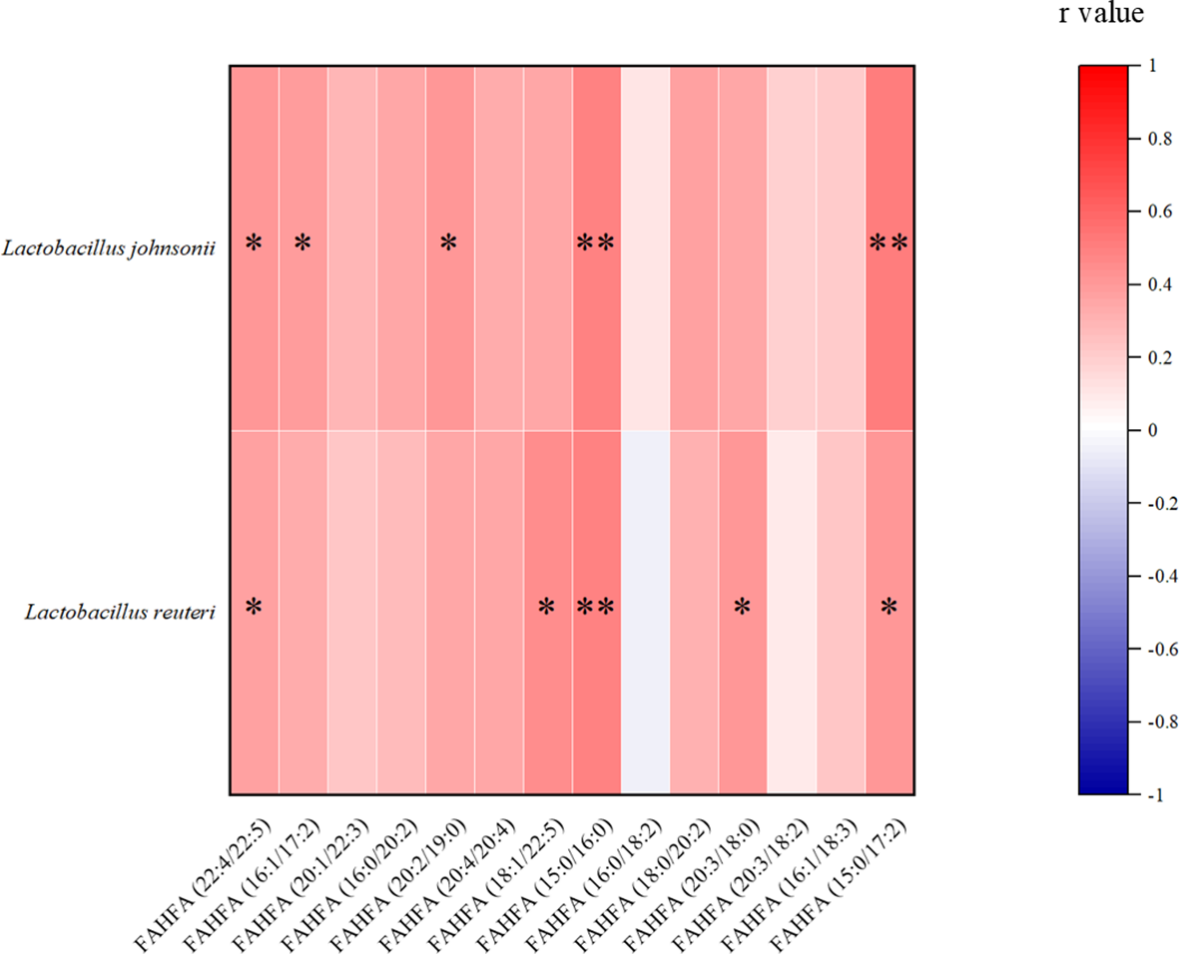
Spearman correlation analysis between FAHFAs levels and *Lactobacillus johnsonii* and *Lactobacillus reuteri.* *p<0.05, **p<0.01.

In our gut metabolomics results, we contrasted the differences between groups for all matched FAHFAs (**Figure 5A**). We selected the FAHFAs that differed significantly in AO vs. CIS (**Figure 5B**). The levels of FAHFA (16:0/20:2), FAHFA (16:0/18:2), FAHFA (18:0/20:2) and FAHFA (20:3/18:2) increased significantly after alginate oligosaccharide intervention compared to that in the CIS group (*p<*0.01). FAHFA (15:0/17:2) level increased significantly after alginate oligosaccharide intervention compared to that in the CIS group (*p<*0.05). We analyzed Spearman correlation between FAHFAs levels and *Lactobacillus johnsonii* and *Lactobacillus reuteri* (**Figure 6**).

### AO Increases Gut FAHFAs Levels Content Through Gut Microbiota and Improves Oxidative Stress Through Nrf2 Pathway

**Figure 7:**
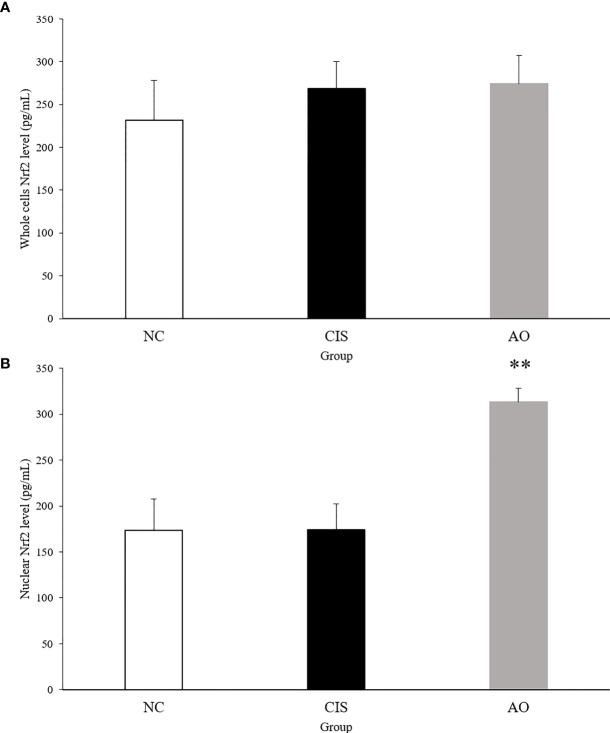
Nrf2 protein level in kidney tissue. **(A)** Whole cell Nrf2 protein level in kidney tissue; **(B)** nuclear Nrf2 protein level in kidney tissue. The significance marker on AO indicates AO vs. CIS (**p<0.01). n=10 for the AO and NC groups, n=7 for the CIS group (three mice in the CIS group died before euthanasia).

NF-E2-related factor 2 (Nrf2) is an important protein involved in oxidative stress response (14). Nrf2 has to be activated to act as an antioxidant function. Activated Nrf2 induces and regulates the expression of a series of downstream antioxidant factors in the nuclear. Therefore, we measured Nrf2 levels in liver and kidney tissues. **Figure 7** shows the level of Nrf2 in the nuclear. ELISA of nuclear Nrf2 in renal tissue showed that the level of nuclear Nrf2 protein in the AO group was significantly higher than that in the NC and CIS groups (*p<*0.01).

## Discussion

Inflammation and oxidative stress caused by cisplatin chemotherapy are often accompanied by acute liver and kidney injury, and even death in severe cases, which is the most important factor currently limiting cisplatin chemotherapy (15). Alginate oligosaccharide has been shown to improve the enteritis and reproductive toxicity caused by the chemotherapeutic drug, busulfan, both *in vivo* and *in vitro *(4, 13). Reports have shown that cisplatin can cause serious liver injury and significant nephrotoxicity (3). Although the mechanism of cisplatin hepatorenal toxicity is not clear, cisplatin is known to play an important role in liver and kidney inflammatory response and oxidative stress. We proposed that alginate oligosaccharide was involved in reducing cisplatin-induced oxidative stress and inflammation *via* intestinal microbiota or its derived metabolites. Our results confirmed that alginate oligosaccharide treatment reduced cisplatin-induced hepatitis and renal redox injury.

Alginate oligosaccharide intervention improves the gut microbiota of mice that had undergone cisplatin chemotherapy. Therefore, we inferred that alginate oligosaccharide improved the side effects of chemotherapy by improving the gut microbiota and its metabolites. *Lactobacillus johnsonii* is the key intestinal strain required for alginate oligosaccharide-mediated protection from cisplatin-induced liver inflammation*. Lactobacillus johnsonii* modulated the biosynthesis of important host metabolites mediating inflammation (16). In porcine mammary epithelial cells, *Lactobacillus johnsonii* L531 inhibited *Escherichia coli*-induced increase in IL-1*β* level (17). Studies have shown that *Lactobacillus johnsonii* significantly enhanced efficacy of immune checkpoint inhibitors in four different mouse models of cancer (18). In our previous review, we described the mechanism and role of *Lactobacillus reutrei* in the phenotype of obesity related inflammation (19). In scurfy mice, *Lactobacillus reuteri* prolonged survival and reduced multiorgan inflammation including liver (20). In LPS-induced inflammation RAW264.7 cells, *Lactobacillus reuteri* reduced the IL-1*β* levels (21). Our results showed that alginate oligosaccharide intervention increased the abundance of *Lactobacillus johnsonii*, indicating that alginate oligosaccharide improved the damage caused by cisplatin and enhanced the effect of cisplatin on cancer treatment.

Alginate oligosaccharide intake increased the content of intestinal fatty acid esters of hydroxy fatty acids (FAHFAs). FAHFAs levels are low in animals, while *Lactobacillus* genus contain enzymes related to the production of hydroxy fatty acids (22), a synthetic substrate of FAHFAs (23). FAHFAs were first identified by Yore et al (24), who demonstrated that activation of GPR120 by FAHFAs reduced the production of IL-1*β* in immune macrophages, thereby alleviating inflammation, which is consistent with our results. In rats with knee osteoarthritis, FAHFAs levels are related to IL-1*β* expression (25). In RAW 264.7 cells, Rachmad et al. confirmed that FAHFAs exhibited anti-inflammatory effect by suppressing LPS-stimulated cytokines, including IL-1*β* and IL-6 (26). This is consistent with the LPS and IL-1*β* level observed in liver tissue after alginate oligosaccharide treatment. We speculated that FAHFAs are obtained by fermentation of intestinal microorganisms and are enriched in the liver and adipose tissue *via* the bloodstream. Alginate oligosaccharide improved cisplatin-induced inflammation by increasing the levels of FAHFAs.

Under normal physiological conditions, Nrf2 bounds to the negative regulatory protein Kelch-like ECH-associated protein 1 (Keap1) in the cytoplasm, which interacts with Nrf2 and acts as an adaptor protein to stabilize the normal condition of Nrf2 (27). When oxidative stress occurs, Keap1 detects oxidative stress through binding of redox sensitive cysteine residues, such as cys151, cys273 and cys288, as well as releases Nrf2 from Keap1 (28). When Nrf2 is transferred to the nucleus, it binds to antioxidant response elements (AREs), which induces the transcription of genes related to cellular oxidoreductases, such as CAT and SOD (29). Therefore, we measured the Nrf2 levels in the nucleus and in whole cells of renal tissue. The AO group compared with the CIS group, the activated Nrf2 level in the nucleus of renal tissue significantly increased and that in whole cells did not have significant difference (**Figure 7**). CAT and SOD can enhance the resistance of cells to oxidative stress (30). In our results, the levels of CAT and SOD in renal tissue increased in the AO group compared with the CIS group (**Figure 1**). MDA is a direct product of lipid peroxidation and is used as an indicator of the severity of oxidative stress (31). In our results, the level of MDA in the AO group decreased compared with the CIS group (**Figure 1**). Therefore, it indicates that the effect of AO administration on renal oxidative stress induced by cisplatin is to activate Nrf2 and then increase antioxidant enzymes (CAT and SOD) levels to resist oxidative stress.

Recently, Siddabasave et al. found that FAHFAs showed less cytotoxicity compared to their native fatty acids and activated Nrf2 in a dose-dependent pattern (32). In neuron-like PC12 cells, alginate oligosaccharide enhanced Nrf2 activation in response to H_2_O_2_-induced endoplasmic reticulum and mitochondrial-dependent apoptotic cell death and oxidative stress (5). In C57BL/6J mice, alginate oligosaccharide ameliorated D-galactose-induced kidney aging in mice *via* activation of the Nrf2 signaling pathway (33, 34). This is consistent with our experimental results. In addition, we found that alginate oligosaccharides might affect FAHFAs levels by altering the abundance of intestinal microorganisms, and that FAHFAs may be an important metabolite of the alginate oligosaccharide-induced Nrf2 pathway *in vivo*.

**Figure 8:**
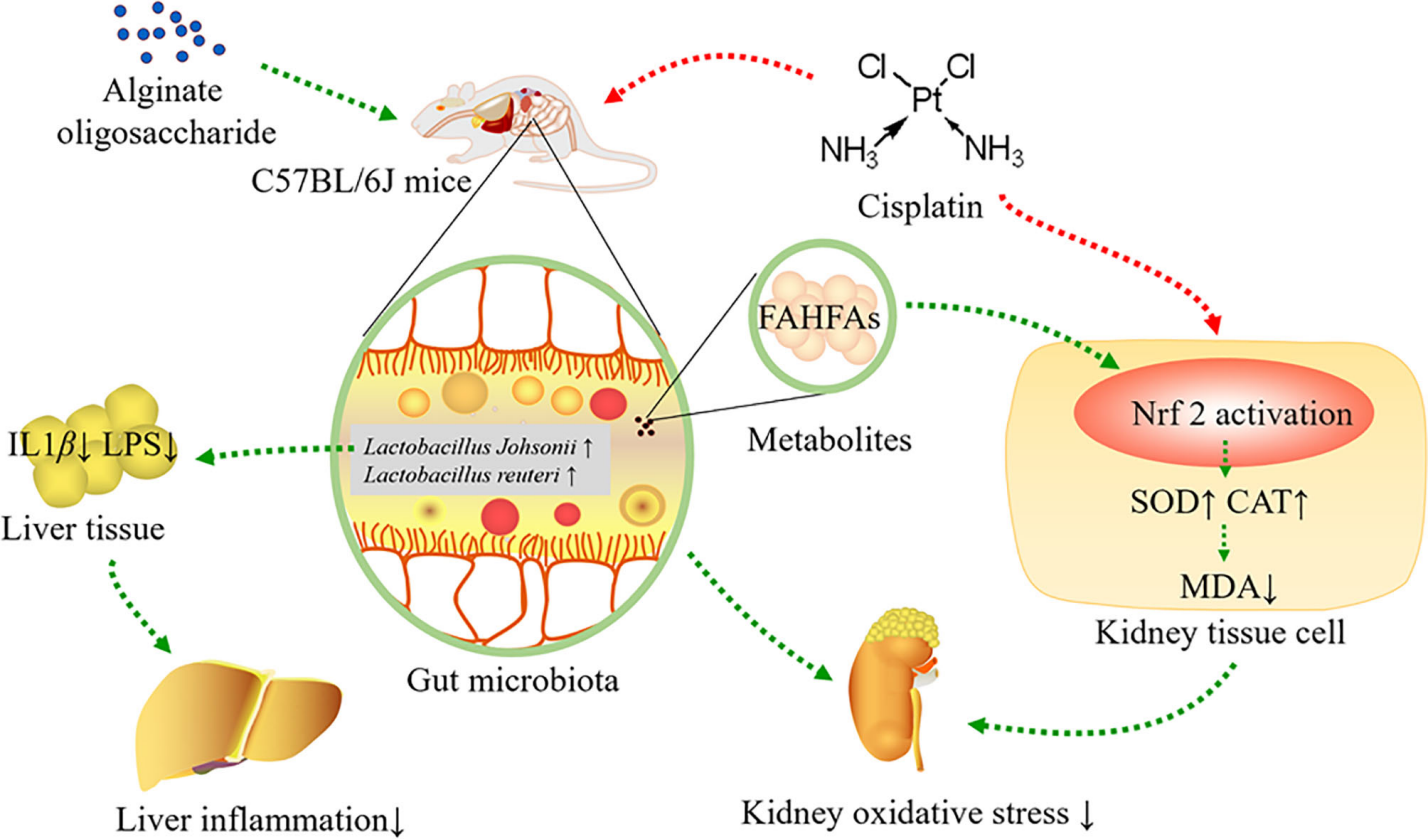
Alginate oligosaccharide alleviated cisplatin-induced kidney oxidative stress *via Lactobacillus* genus-FAHFAs-Nrf2 axis in mice.

In conclusion, alginate oligosaccharide increased the levels of *Lactobacillus johnsonii* and *Lactobacillus reuteri*, as well as bacterial related metabolite-FAHFAs levels, improved kidney redox injury caused by cisplatin chemotherapy, including enhancing the levels of SOD and CAT in kidney tissue, as well as reducing MDA levels in kidney *via* stimulating Nrf2. Furthermore, alginate oligosaccharide ameliorated liver inflammation by decreasing the levels of LPS and IL-1*β* in liver tissue (**Figure 8**).

## Data Availability Statement

The data presented in the study are deposited in the NCBI repository, accession number PRJNA779197. The link is https://www.ncbi.nlm.nih.gov/bioproject/779197.

## Ethics Statement

The animal study was reviewed and approved by Yantai Yuhuangding hospital ethics committee.

## Author Contributions

LL designed the experiment. YZ and JY carried out the experiment. YZ wrote this manuscript. YS and SH given the experimental environment and equipment support. LL and SQ given the financial support and revised the manuscript. YZ and MC analyzed the data. All authors contributed to the article and approved the submitted version.

## Funding

This work was supported by Youth Innovation Promotion Association of Chinese Academy of Sciences (2018246) and Science and Technology Program of Yantai (2020MSGY076).

## Conflict of Interest

The authors declare that the research was conducted in the absence of any commercial or financial relationships that could be construed as a potential conflict of interest.

## Publisher’s Note

All claims expressed in this article are solely those of the authors and do not necessarily represent those of their affiliated organizations, or those of the publisher, the editors and the reviewers. Any product that may be evaluated in this article, or claim that may be made by its manufacturer, is not guaranteed or endorsed by the publisher.
